# 免疫检查点抑制剂相关输尿管膀胱炎：病例报道1例及文献复习

**DOI:** 10.3779/j.issn.1009-3419.2023.106.17

**Published:** 2023-09-20

**Authors:** Shishi LI, Ke ZHENG, Yan XU, Mengzhao WANG

**Affiliations:** ^1^100730 北京，中国医学科学院，北京协和医学院，北京协和医院呼吸与危重症医学科; ^1^Department of Respiratory and Critical Care Medicine, Peking Union Medical College Hospital, Chinese Academy of Medical Sciences and Peking Union Medical College, Beijing 100730, China; ^2^410300 长沙，浏阳市中医医院呼吸与危重症医学科; ^2^Department of Respiratory and Critical Care Medicine, Liuyang Hospital of Chinese Medicine, Changsha 410300, China; ^3^100730 北京，中国医学科学院，北京协和医学院，北京协和医院肾内科; ^3^Department of Nephrology, Peking Union Medical College Hospital, Chinese Academy of Medical Sciences and Peking Union Medical College, Beijing 100730, China

**Keywords:** 免疫检查点抑制剂, 免疫相关不良反应, 肺肿瘤, 输尿管膀胱炎, 急性肾损伤, Immune checkpoint inhibitors, Immune-related adverse events, Lung neoplasms, Cystoureteritis, Acute kidney injury

## Abstract

1例应用帕博利珠单抗联合化疗治疗的晚期肺腺癌患者在治疗14个周期后出现了尿频、尿急症状。经尿常规、肾功能、膀胱镜及计算机断层扫描（computed tomography, CT）检查考虑为免疫抑制剂相关性输尿管膀胱炎以及急性肾损伤。停用帕博利珠单抗联合化疗后症状缓解，再次应用帕博利珠单抗联合化疗尿路刺激症状明显加重，应用激素治疗后症状缓解。在使用免疫检查点抑制剂时，患者如出现泌尿系统症状，需考虑免疫相关输尿管膀胱炎，尽早识别和治疗。

## Abstract (en)

A patient with advanced lung adenocarcinoma developed symptoms of frequent urination and urgent urination after 14 cycles of Pembrolizumab combined with chemotherapy. After making comprehensive analysis of the results of urine routine test, renal function, cystoscope and computed tomography (CT) examination, immune checkpoint inhibitors related cystoureteritis and acute kidney injury were considered. The patient’s symptoms were relieved after discontinuation of Pembrolizumab combined with chemotherapy. However, the symptoms of urinary irritation worsened significantly after rechallenging Pembrolizumab combined with chemotherapy, and the symptoms was relieved after corticosteroids treatment. If patients develop urinary symptoms during immune checkpoint inhibitors treatment, immune checkpoint inhibitors related cystoureteritis should be considered for early differential diagnosis in order to implement appropriate treatment.

肺癌在全世界男性癌症死因中居首位，在女性癌症死因中居第2位，2018年全世界约有210万例新发肺癌，约180万例死亡^[1]^。免疫检查点抑制剂（immune checkpoint inhibitors, ICIs）的应用，给肺癌患者带来显著生存获益^[2]^。随着ICIs在晚期肺癌患者中的广泛应用，ICIs所导致的免疫相关不良反应（immune-related adverse events, irAEs）也越来越受到重视。由于ICIs通过增强免疫抗肿瘤作用来消除肿瘤细胞，它们也可能导致免疫过度激活，从而攻击正常组织，导致irAEs^[3]^。在中国，患者因程序性死亡受体1/程序性死亡受体配体-1（programmed cell death 1/programmed cell death ligand 1, PD-1/PD-L1）单抗治疗肺癌所导致的irAEs以内分泌系统、肺和皮肤中观察到的器官特异性irAEs发生率高，分别为8.3%、6.7%和6.0%^[4]^。另有罕见的irAEs，如神经系统或关节损害，发生率小于1%^[4]^。免疫相关性泌尿系统不良反应常见报道的为肾脏损害，发病率约为1%，而输尿管炎、膀胱炎很少报道，累及整个泌尿系统的病例则更少^[4]^。本文报道1例帕博利珠单抗联合化疗治疗后出现膀胱炎、输尿管炎和急性肾损伤的病例。

## 1 病例资料

患者男，57岁，因胸部隐痛3月就诊，胸部增强电子计算机断层扫描（computed tomography, CT）示左肺上叶、下叶不规则团片影，纵隔4R/L、6、7区多发淋巴结，左肺中-大量胸腔积液。胸腔镜胸膜活检支持肺腺癌，免疫组化：D2-40（podoplanin）（-），黑皮质素（melanocortin, MC）（-），癌症危险基因（cancer risk, CR）（±），天冬氨酸蛋白酶（novel aspartic proteinase A, Napsin A）（+），Wilms肿瘤1（Wilm tumor gene 1, WT1）（-），细胞角蛋白5/6（cytokeratin 5/6, CK5/6）（-），增殖细胞相关抗原（nuclear associated antigen 67, Ki-67）（20%+），波形蛋白（Vimentin）（-），细胞角蛋白单克隆抗体1/3（cytokeratin monoclonal antibodies 1/3, AE1/AE3）（3+），甲状腺转录因子1（thyroid transcription factor-1, TTF-1）（+）。由于肿瘤组织样本不足，故行外周血循环肿瘤DNA基因检测（第二代测序技术）未发现驱动基因突变。患者诊断为肺腺癌，分期T4N3M1a，IVA期。患者既往体健，否认肾炎、膀胱炎、前列腺增生、自身免疫性疾病病史；有大量吸烟史；其母亲有直肠癌病史。基线血肌酐为47 μmol/L。2021年6月3日起，患者开始接受帕博利珠单抗200 mg d1联合培美曲塞950 mg d1联合卡铂600 mg d1治疗，每21天一次，共4个周期。疗效评估病灶稳定。2021年9月3日起，患者帕博利珠单抗200 mg d1联合培美曲塞950 mg d1维持治疗，每21天一次，共10个周期，治疗过程中无明显不良反应。定期疗效评估疾病稳定。患者于2022年4月出现尿频、尿急，无畏寒、寒战、发热。流式尿沉渣分析显示白细胞（white blood cell, WBC）为208.9/μL（0-8.6）/μL，红细胞（red blood cell, RBC）为2.0/μL（0-8.4）/μL，尿培养阴性，血肌酐为62 μmol/L，泌尿系超声未见明显异常。当地医院给予头孢地尼抗感染，约10 d后症状缓解，继续第15-16个周期培美曲塞联合帕博利珠单抗治疗。疗效评价病灶稳定。在此期间均在用药后5-7 d出现尿频、尿急、下腹紧缩样痛、右侧腰痛伴排尿中存在絮状、膜状物，排尿时加重、尿后疼痛有缓解，无发热，无尿量减少，无恶心、呕吐等不适，抗感染疗效欠佳，但在10-15 d后泌尿系统症状可消退。2022年6月行第17个周期帕博利珠单抗联合培美曲塞治疗，出现血尿、耻骨上疼痛。再次尿培养阴性。CT尿路造影（CT urography, CTU）提示：双侧肾盂肾盏及输尿管管壁增厚、毛糙，可见强化，双侧输尿管周围多发索条影；与基线（图1）对比，膀胱壁增厚、毛糙，可见轻中度强化（图2）。膀胱镜检查未见明显异常。考虑不除外ICIs相关不良反应可能。此后患者暂停抗肿瘤治疗，休疗期间尿路刺激症状未再发作。2022年9月26日复查胸部CT胸水增多，考虑为肺癌进展，2022年10月11日重启培美曲塞联合帕博利珠单抗治疗，2022年10月19日再次出现尿频、尿急、小腹部胀痛，无恶心、呕吐、少尿等不适，尿培养阴性，流式尿沉渣分析显示RBC为57.5/μL、变形RBC比例为0，WBC为704.8/μL，血肌酐为100 μmol/L，泌尿系超声仍正常。结合影像学检查、膀胱镜检查结果，尿培养多次无菌生长，考虑帕博利珠单抗相关泌尿系不良反应：输尿管膀胱炎[常见不良事件评价标准（Common Terminology Criteria for Adverse Events, CTCAE）2级]、急性肾损伤（CTCAE 2级）。因患者临床症状明显，给予中等剂量激素治疗，2022年10月19日开始泼尼松30 mg，每日一次，口服治疗，次日症状明显减轻。予泼尼松30 mg，每日一次，维持2周后规律减量，每周减量5 mg至减停。2023年1月4日复查尿常规显示WBC为7.1/μL，RBC为392.9/μL，血肌酐为76 μmol/L，未再出现尿路刺激症状，腹部CT示肾盂、输尿管、膀胱壁增厚较前好转（图3）。2023年3月随访患者无尿路刺激症状、无腹痛，每日尿量1500-2000 mL，无胸闷、胸痛、气促、咳嗽等不适。本研究通过中国医学科学院北京协和医院伦理审查（No.K2135），并获得患者知情同意。

**图1:**
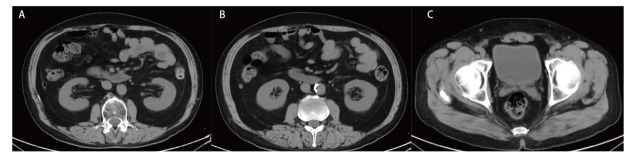
2021年9月2日腹盆CT平扫未见明显泌尿系病变的肾盂肾盏（A）、输尿管（B）、膀胱（C）基线情况。

**图2:**
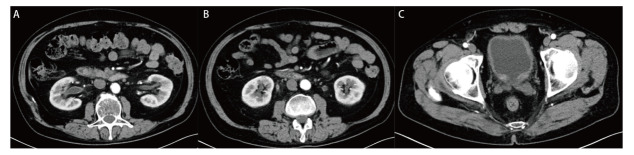
帕博利珠单抗联合化疗治疗17个周期之后出现irAEs时，2022年6月17日CT尿路成像示双侧肾盂肾盏管壁增厚、毛糙，可见强化（A），双侧输尿管壁增厚、毛糙，强化周围可见多发索条影（B），膀胱壁增厚、毛糙，可见轻中度强化（C）。

**图3:**
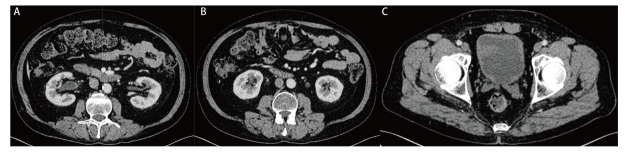
2022年12月5日腹盆增强CT示激素治疗后双侧肾盂管壁（A）、输尿管管壁（B）、膀胱壁（C）增厚较前好转。

## 2 讨论

本例病例中使用帕博利珠单抗联合化疗过程中出现尿路刺激症状，症状与用药明显相关，停药期间无发作，再次使用帕博利珠单抗联合培美曲塞治疗，尿路刺激症状再次发生，反复尿培养未见细菌生长，膀胱镜检查排除出血、肿瘤、结石等，CTU可见明显肾盂肾盏管壁、输尿管管壁、膀胱壁均出现增厚、毛躁、强化，反复抗感染疗效欠佳，激素治疗后明显缓解。结合影像学考虑输尿管膀胱炎，因患者临床症状明显，评估为CTCAE 2级。此外，患者血肌酐较前升高超过2倍，CTCAE 2级，考虑存在急性肾损伤，无肾前性因素，无排尿困难等尿路梗阻表现，超声无肾盂显著扩张，排除肾后性因素，故肾性可能大，尿常规提示尿WBC升高，RBC增高不明显，无明显蛋白尿，结合ICIs肾脏受累的特点，考虑间质性肾炎可能性大。治疗反应支持诊断。本病例可见膀胱输尿管炎的尿路上皮炎症表现以及急性肾损伤，考虑为累及泌尿系统的irAEs。

目前文献^[5]^报道的泌尿系统irAEs常见的为肾脏irAEs，包括急性间质性肾炎、急性肾小管坏死和较少见的肾小球疾病，较少有膀胱炎、输尿管炎等尿路上皮炎症报道。针对尿路上皮的炎症反应，我们认为输尿管炎、膀胱炎为尿路上皮相关不良反应，可定义为ICIs相关输尿管膀胱炎，为ICIs相关尿路上皮炎。通过检索（检索关键词：免疫检查点抑制剂和输尿管炎、膀胱炎或尿道炎）截至2023年7月3日的PubMed、SpringerLinK、万方数据、中国知网、维普记录文献复习见表1^[6⇓⇓⇓⇓⇓⇓⇓⇓⇓⇓-17]^。免疫抑制剂相关尿路上皮炎发生的年龄阶段为47岁-78岁不等，肺癌患者有10例，肝内胆管癌1例，乳腺癌1例，黑色素瘤1例，食管癌1例，胃癌2例，输尿管炎5例，膀胱炎16例，可能对于输尿管炎重视度不足、评价方式有限，导致报道极少。纳武利尤单抗相关9例、帕博利珠单抗相关3例、阿替利珠单抗1例、信迪利单抗2例、伊匹木单抗1例、替雷丽珠单抗1例。发生在使用免疫抑制剂第2-77个周期，患者主要临床表现为尿频、尿急、尿痛、血尿，14例尿培养阴性，2例可见细菌，尿常规均有白细胞增多，提示无菌性膀胱炎。大部分膀胱镜可见膀胱黏膜发红。膀胱病理主要为T淋巴细胞浸润为主，1例可见中性粒细胞浸润。14例经激素治疗迅速缓解，2例停药后自行缓解。由此可见在这些发生免疫相关不良反应的病例中，患者均在使用ICIs后发生尿频、尿急、尿痛、血尿等相关泌尿系症状。尿常规均有白细胞增多，但尿培养为阴性，膀胱活检病理均未见肿瘤细胞，大部分以T淋巴细胞浸润为主，经激素治疗或停药治疗后泌尿系统症状均明显好转。目前发生的机制不明确，推测可能的机制为T细胞参与膀胱炎有关，T细胞产生细胞因子作用于尿路上皮，导致尿路上皮炎。目前可预测irAEs风险且有助于早期识别这些并发症的最佳生物标志物仍有待确定，影像学评估可以作为参考依据。

**表1:** 文献报道免疫相关性尿路上皮炎患者资料

Case		Sex	Age (yr)	Tumor histology	Onset time	Clinical signs and symptoms	Urine routine	Uroeti-ology	Cystoscopic results	Pathological findings	Imageo- logical examination	Urinary tract involvement	Treatment
1^[6]^		Male	50	Squamous cell carcinoma of lung	After 7 cycles of Nivolumab	Pollakisuria, micturition pain, and diarrhea	WBC: >100/HPF	Negative	Undone	Undone	Undone	Bladder	Prednisone 60 mg for immediate relief
2^[6]^		Male	60	Squamous cell carcinoma of lung	After 12 courses of Nivolumab	Pollakisuria, dysuria, and diarrhea	Pyuria (neutrophil and lymphocyte composition)	Negative	Undone	Undone	Undone	Bladder	Discontinuing Nivolumab treatment
3^[7]^		Male	62	Squamous cell carcinoma of lung	After 3 courses of Nivolumab	Fever, diarrhea, frequent urination, pain in urine, grossematuria	RBC: >100/HPF; WBC: 5-9/HPF	Negative	Diffuse redness and erosion of the bladder mucosa	Epithelial desquamation and edematous changes in interstitium were observed	No abnor- malities	Bladder	Methylprednisolone 500 mg for 3 days, Prednisone 0.5 mg/kg tapering
4^[8]^		Female	78	Lung adenocar-cinoma	After 6 cycles of Pembro-lizumab	Pollakiuria and nocturia accompanied by painful micturition	RBC: >100/HPF; WBC: >100/HPF	Negative	The bladder mucosa is red and edema	The urothelium strongly expressed PD-L1 but did not show significant atypia suggesting malignancy. PD-L1-positive cells were also found in the subepithelial tissue. These cells were presumed to be histiocytes. Infiltrates of CD8-positive and/or TIA-1-positive lymphocytes are present in the epithelium.	Undone	Bladder	The patient’s subjective symptoms and findings on cystoscopy improved dramatically after 19 days of treatment with Prednisolone 25 mg/d
5^[9]^		Male	60	Lung cancer	After 77 courses of Nivolumab	Glans penile pain and micturition pain	Pyuria	Negative	Bladder mucosa diffuse redness	Undone	No abnormalities	Bladder	Methylprednisolone 60 mg tapered
6^[10]^		Male	53	Lung adenocar-cinoma	After 3 courses of Sintilimab	Hematuria, frequent urination, pain in urine, lumbago	RBC: 3889.7/μL; WBC: 2133.5/μL	Negative	Bladder mucosa diffuse redness	Lymphocytic inflammation and interstitial tissue hyperplasia	Urinary ultrasonography showed mild hydronephrosis and dilated ureter	Bladder and ureter	Methylprednisolone 80 mg was gradually reduced
Case		Sex	Age (yr)	Tumor histology	Onset time	Clinical signs and symptoms	Urine routine	Uroeti-ology	Cystoscopic results	Pathological findings	Imageo- logical examination	Urinary tract in-volvement	Treatment
7^[11]^		Male	48	Intrahepatic bile duct carcinoma	After 3 courses of Nivolumab	Urinary tract irritation symptoms	WBC: 2818/μL	Bacteria: 512/μL	NA	Chronic inflammation of mucosal tissue, mucosal erosion in some areas, and proliferation of granulation tissues and fibroblasts	Undone	Bladder	Glucocorticoid 2 mg/kg
8^[12]^		Male	51	Small cell lung cancer	After 5 courses of Nivolumab	Urgent urination, difficulty urinating	Neutrophils and lymphocytes	Negative	Mucosal edema	CD3 and CD8-positive lymphocytes infiltrate the urothelium	Undone	Bladder	Methylprednisolone 80 mg was tapered
9^[13]^		Female	61	Melanoma	After 4 courses of Nivolumab and Ipilimumab	Diarrhea, frequent urination, pain in urine	WBC increased	Negative	The bladder mucosa is red and swollen	Lymphocyte T cell infiltration in intraepithelial and subepithelial connective tissue	Undone	Bladder	Prednisolone 0.5 mg/kg/d
10^[14]^		Male	47	Pulmonary adenocar- cinoma	After 18 courses of Nivolumab	Frequent and painful urination	WBC: ≥100/HPF	Small number of bacteria	Hemorrhages were seen from the bladder wall after expansion of the bladder	Slightly strong inflammatory cell infiltration mainly composed of eosinophils and plasma cells were observed, and some eosinophils showed degranulation. There was no evidence of malignancy	CT scan of the abdomen: thickening of the bladder wall	Bladder	The symptoms disappeared after biopsy
11^[15]^		Female	67	Breast cancer	After 97 days of Atezolizumab	Frequent urination, painful urination	NA	Negative	Diffuse redness of bladder mucosa	Histopathological examination showed no evidence of malignancy and the absence of inclusion bodies in the epithelium	Undone	Bladder	Prednisone 1 mg/kg
12^[16]^		Male	56	Squamous cell carcinoma of lung	6^th^ cycle of Pembrolizumab	Frequent, urgent and painful urination	RBC: >60/μL; WBC: >100/μL	Negative	NA	This showed lymphocytes and many neutrophils clustered into a microscopic abscess. Immunohistochemical analysis revealed a large number of CD8 T cells and TIA-1 lymphocytes infiltrated	Undone	Bladder	Methylprednisolone 40 mg×3 days
Case		Sex	Age (yr)	Tumor histology	Onset time	Clinical signs and	Urine routine	Uroeti-ology	Cystoscopic results	Imageo- logical examination	Imageo- logical examination	Urinary tract in-volvement	Treatment
13^[17]^		Male	49	Esophageal carcinoma	After six courses of Tislelizumab	Gross hematuria, pollakiuria, painful micturition, and low back pain	RBC: 4932/μL; WBC: 9375/μL; proteinuria 3+	Negative	Diffused redness of the bladder mucosa	Effacement of the bladder urothelium, hyperplastic granulation tissue, and infiltration of monocytes, lymphocytes, plasmacytes, and neutrophils in the bladder tissue. Immunohistochemistry staining of the bladder tissue showed positive staining of CD3, CD8, CD20, and CD117, yet negative staining of CD68, TIA-1, and PD-L1 in focal lesions	Urinary ultrasonography and computed tomography showed mild hydronephrosis, dilated ureter, and thickened bladder wall	Bladder, ureter	1.5 mg/kg/d of Prednisone
14^[17]^		Female	62	Stage IV gastric carcinoma	After 3 cycles of Sintilimab	Urinary irritation	RBC: 42/μL; WBC: 17,916/μL; proteinuria 3+	Negative	NA	NA	Urinary ultrasonography and CT showed mild hydronephrosis and dilation of the ureter on the left and a thickened bladder wall	Bladder, ureter	1.7 mg/kg/d of Prednisone
15^[17]^		Male	49	Gastric carcinoma	After the 2^nd^ course of Nivolumab	Hematuria, pollakiuria, painful micturition, and fever	RBC: 13,298/μL; WBC: 2506/μL; proteinuria 3+	Negative	NA	NA	Urinary ultrasonography and CT showed mild hydronephrosis, dilated ureters, and thickened bladder wall	Bladder, ureter	1.7 mg/kg/d of Prednisone
16 (Current patient)		Male	57	Lung adenocar-cinoma	After 14 cycles of Pembrolizumab	Frequent urination, urgent urination, lower abdominal pain, hematuria	RBC: 57.5/μL; WBC: 704.8/μL	Negative	No abnormality found	NA	The walls of the renal pelvis and caliceal ducts, ureter and bladder were thickened and coarse	Bladder, ureter, kidney	Prednisone 30 mg

WBC: white blood cells; HPF: high power field; RBC: red blood cell; PD-L1: programmed cell death ligand 1; CT: computed tomography; TIA-1: T-cell restricted intracellular antigen-1; NA: not applicable.

结合该病例和文献复习，提示使用ICIs的患者如出现尿频、尿急、尿痛尿路刺激症状，反复抗感染治疗无效，需要考虑ICIs相关尿路上皮炎不良反应的可能性，通过影像学检查及内镜检查定位于输尿管及膀胱。此类患者需要密切关注泌尿系统症状及尿量变化，及时检查尿常规和尿培养，完善血液学检查，行影像学评估，必要时行膀胱镜检查。在排除泌尿系感染、肿瘤等可能后，可考虑ICIs相关尿路上皮炎，此类患者可同时合并肾脏irAEs。因此类患者临床表现和相关检查特异性差，以排除性诊断为主。如考虑泌尿系统irAEs，可停药观察病情变化，必要时可给予糖皮质激素治疗。

## 3 总结

对于使用ICIs的患者，出现尿频、尿急、血尿、腹痛症状时，需要完善肾功能、尿液检查，并及时完善影像学评估，除外泌尿系感染后应考虑ICIs相关尿路上皮炎（输尿管炎、膀胱炎等），并需要依据影像学进行进一步的解剖定位，此类患者也可同时合并肾脏损害，如症状反复或持续，必要时给予激素治疗。

Conflicts of interest

The authors have no conflicts of interest to declare.

## References

[1] Ferlay J, Colombet M, Soerjomataram I, et al. Estimating the global cancer incidence and mortality in 2018: GLOBOCAN sources and methods. Int J Cancer, 2019, 144(8): 1941-1953. doi: 10.1002/ijc.31937 30350310

[2] Reck M, Rodriguez-Abreu D, Robinson AG, et al. Pembrolizumab versus chemotherapy for PD-L1-positive non-small cell lung cancer. N Engl J Med, 2016, 375(19): 1823-1833. doi: 10.1056/NEJMoa1606774 27718847

[3] Yoest JM. Clinical features, predictive correlates, and pathophysiology of immune-related adverse events in immune checkpoint inhibitor treatments in cancer: a short review. Immunotargets Ther, 2017, 6: 73-82. doi: 10.2147/ITT.S126227 29067284 PMC5644546

[4] Shi Y, Fang J, Zhou C, et al. Immune checkpoint inhibitor-related adverse events in lung cancer: Real-world incidence and management practices of 1905 patients in China. Thorac Cancer, 2022, 13(3): 412-422. doi: 10.1111/1759-7714.14274 34935288 PMC8807338

[5] Meraz-Munoz A, Amir E, Ng P, et al. Acute kidney injury associated with immune checkpoint inhibitor therapy: incidence, risk factors and outcomes. J Immunother Cancer, 2020, 8(1): e000467. doi: 10.1136/jitc-2019-000467 32601079 PMC7326260

[6] Shimatani K, Yoshimoto T, Doi Y, et al. Two cases of nonbacterial cystitis associated with nivolumab, the anti-programmed-death-receptor-1 inhibitor. Urol Case Rep, 2018, 17: 97-99. Doi 10.1016/j.eucr.2017.12.006 29541592 PMC5849865

[7] Ozaki K, Takahashi H, Murakami Y, et al. A case of cystitis after administration of nivolumab. Int Cancer Conf J, 2017, 6(4): 164-166. doi: 10.1007/s13691-017-0298-6 31149494 PMC6498356

[8] Ueki Y, Matsuki M, Kubo T, et al. Non-bacterial cystitis with increased expression of programmed death-ligand 1 in the urothelium: An unusual immune-related adverse event during treatment with pembrolizumab for lung adenocarcinoma. IJU Case Rep, 2020, 3(6): 266-269. doi: 10.1002/iju5.12211 33163921 PMC7609190

[9] Fukunaga H, Sumii K, Kawamura S, et al. A case of steroid-resistant cystitis as an immune-related adverse event during treatment with nivolumab for lung cancer, which was successfully treated with infliximab. IJU Case Rep, 2022, 5(6): 521-523. doi: 10.1002/iju5.12532 36341187 PMC9626353

[10] Tu L, Ye Y, Tang X, et al. Case report: A case of sintilimab-induced cystitis/ureteritis and review of sintilimab-related adverse events. Front Oncol, 2021, 11: 757069. doi: 10.3389/fonc.2021.757069 35004277 PMC8733470

[11] Zhu S, Bian L, Lv J, et al. A case report of non-bacterial cystitis caused by immune checkpoint inhibitors. Front Immunol, 2021, 12: 788629. doi: 10.3389/fimmu.2021.788629 35003107 PMC8733335

[12] Zhu L, Wang Z, Stebbing J, et al. Immunotherapy-related cystitis: case report and review of the literature. Onco Targets Ther, 2021, 14: 4321-4328. doi: 10.2147/OTT.S321965 34366676 PMC8336986

[13] Schneider S, Alezra E, Yacoub M, et al. Aseptic cystitis induced by nivolumab and ipilimumab combination for metastatic melanoma. Melanoma Res, 2021, 31(5): 487-489. doi: 10.1097/CMR.0000000000000765 34433197

[14] Yajima S, Nakanishi Y, Matsumoto S, et al. Improvement of urinary symptoms after bladder biopsy: A case of pathologically proven allergy-related cystitis during administration of nivolumab. IJU Case Rep, 2021, 4(4): 213-215. doi: 10.1002/iju5.12286 34308272 PMC8294138

[15] Obayashi A, Hamada-Nishimoto M, Fujimoto Y, et al. Non-bacterial cystitis with increased expression of programmed cell death ligand 1 in the urothelium: An unusual immune-related adverse event after Atezolizumab administration for metastatic breast cancer. Cureus, 2022, 14(5): e25486. doi: 10.7759/cureus.25486 35800819 PMC9246443

[16] He X, Tu R, Zeng S, et al. Non-bacterial cystitis secondary to pembrolizumab: A case report and review of the literature. Curr Probl Cancer, 2022, 46(4): 100863. doi: 10.1016/j.currproblcancer.2022.100863 35687965

[17] Li J, Yu YF, Qi XW, et al. Immune-related ureteritis and cystitis induced by immune checkpoint inhibitors: Case report and literature review. Front Immunol, 2023, 13: 1051577. doi: 10.3389/fimmu.2022.1051577 36685488 PMC9853439

